# The complete chloroplast genome of *Zanthoxylum stenophyllum* Hemsl. (Rutaceae), a traditional Chinese medicinal plant

**DOI:** 10.1080/23802359.2022.2097895

**Published:** 2022-09-15

**Authors:** Qinqin Huang, Xia Liu, Chong Sun, Haowen Liu, Houlin Zhou, Fengting Huang, Han Liu, Zexiong Chen

**Affiliations:** a College of Biology and Food Engineering, Chongqing Three Gorges University, Chongqing, China; b College of Landscape Architecture and Life Science, Chongqing University of Arts and Sciences, Chongqing, China; c Spice Crops Research Institute, College of Horticulture and Gardening, Yangtze University, Jingzhou, China; d Chongqing Wulipo National Nature Reserve Management Office, Chongqing, China

**Keywords:** *Zanthoxylum stenophyllum*, complete chloroplast genome, phylogenetic analysis

## Abstract

*Zanthoxylum stenophyllum* Hemsl., a type species for the genus *Zanthoxylum* (Rutaceae), is a traditional medicinal plant. We studied the complete chloroplast genome of this species using BGISEQ-500 platform. The chloroplast genome was 158,314 bp in size with a GC content of 38.45%. The genome contained two short inverted repeat (IRa and IRb) regions of 27,052 bp, a large single-copy region (LSC, 86,029 bp) and a small single-copy region (SSC, 18,181 bp). The annotated complete chloroplast genome contains 133 distinct genes, including 88 protein-coding genes, 37 transfer RNAs (tRNAs), and 8 ribosomal RNAs (rRNAs). Phylogenetic analysis indicated that *Z. stenophyllum* is clustered with *Z. schiniflium* and *Z. pinnatum* in the same branch with 100% bootstrap support. This complete chloroplast genome provides valuable genomic information for the molecular phylogeny and sustainable utilization of *Zanthoxylum*.

*Zanthoxylum* L. of the family Rutaceae comprises approximately 225 species, most of which have high medicine and economic value (Appelhans et al. 2014). These species are distributed in tropical and subtropical areas of the northern hemisphere (Kubitzki et al. 2011). The genus *Zanthoxylum* reaches the limits of northern Japan, Korea and northern, northwestern and southwestern China (Zhang et al. 2008), where it is well known for its economic importance.

In 1895, W. Botting Hemsley, F. R. S. first published a description of *Zanthoxylum stenophyllum* Hemsl. as a new species (Hemsley 1895). *Zanthoxylum stenophyllum* Hemsl. is a traditional medicine plant in southwestern China and is mainly distributed in the 1000–2200 m mountain forest areas of Shaanxi, Gansu, Sichuan, and western Hubei Provinces of China (Huang 1997). The peel is a raw material used in essence and fragrance, and the seed provides an excellent woody oil; its root bark is used as a medicine to treat traumatic injuries in Hubei, China (Huang et al. 1991). However, only a few studies have investigated phylogenetic relationships in the genus *Zanthoxylum* based on a few chloroplast DNA fragments (Appelhans et al. 2018). To our knowledge, there is no genomic information of *Z. stenophyllum* that has been reported thus far. Thus, in this study, the chloroplast genome of *Z. stenophyllum* was sequenced, assembled and analyzed with related species, and it will provide valuable genomic information for the molecular identification, phylogeny and utilization of germplasm resources.

Fresh *Z*. *stenophyllum* samples were collected from Wushan, Chongqing, China (31°03'21′' N, 110°01'41′' E). The voucher specimen (CUAS-XY01) and extracted DNA were also laid in the Herbarium of Chongqing University of Arts and Sciences (collected by Xia Liu, liuxiavip8@163.com). DNA extraction was isolated following a modified CTAB method (Doyle 1987). Paired-end (150 bp) sequencing was performed by Guangzhou Bio&Data Biotechnologies CO., Ltd. (Guangzhou, China) on the BGISEQ-500 platform. We used the software metaSPAdes (Nurk et al. 2017) to assemble chloroplast genomes. Gene annotation of *Z*. *stenophyllum* was performed using CpGAVAS (Shi et al. 2019), then Geneious 8.0.2 (Campos et al. 2016) was used to correct the results.

The length of the chloroplast genome of *Z*. *stenophyllum* (GenBank accession number MW602896) reaches 158,314 bp, and the GC content of the plastid genome accounts for 38.45% of the total. There were two groups of repeated sequence (IRs, 27,052bp), in which GC content accounts for 41.1%. There are two unique sequences regions, large single copy (LSC, 87,486) and small single copy (SSC, 15,577 bp), 29.7% and 33.8% of the GC content in SSC and LSC, respectively. Annotated whole plastid genome contains 133 genes, comprising 88 protein-coding genes, 37 tRNA and 8 rRNA. There were two exons observed in 19 genes (*trnG-UCC*, *trnK-UUU*, *rpoC1*, *petB*, *trnL-UAA*, *trnV-UAC*, *rpl16*, *petD*, *trnl-GAU*, *trnA-UGC*, *trnA-UGC*, *ndhA*, *atpF*, *trnl-GAU*, *rps16*, two *rpl2* and two *ndhB*) with two exons and contain three exons were observed in 4 genes (*clpP*, *ycf3* and two *rps12*).

To verify the phylogenetic relationships of *Z*. *stenophyllum* with its closely related species, a phylogenetic tree was constructed based on the plastid genome of 12 species with available data (Figure 1). The sequence alignment tool, MAFFT (Katoh and Standley 2013), was used to multiple alignment. The phylogenetic tree was performed by RAxML 8.1.5 (Stamatakis 2014) with 1000 bootstrap. The ML tree showed that *Z*. *stenophyllum* is closely associated with *Z. schinifoliun* and clustered with *Z. pinnatum* in the same branch with 100% bootstrap support. This published *Z*. *stenophyllum* chloroplast genome will provide useful bioinformatics for the molecular identification of closely related species of *Zanthoxylum* and for phylogenetic and evolutionary studies of Rutaceae.

**Figure 1.**
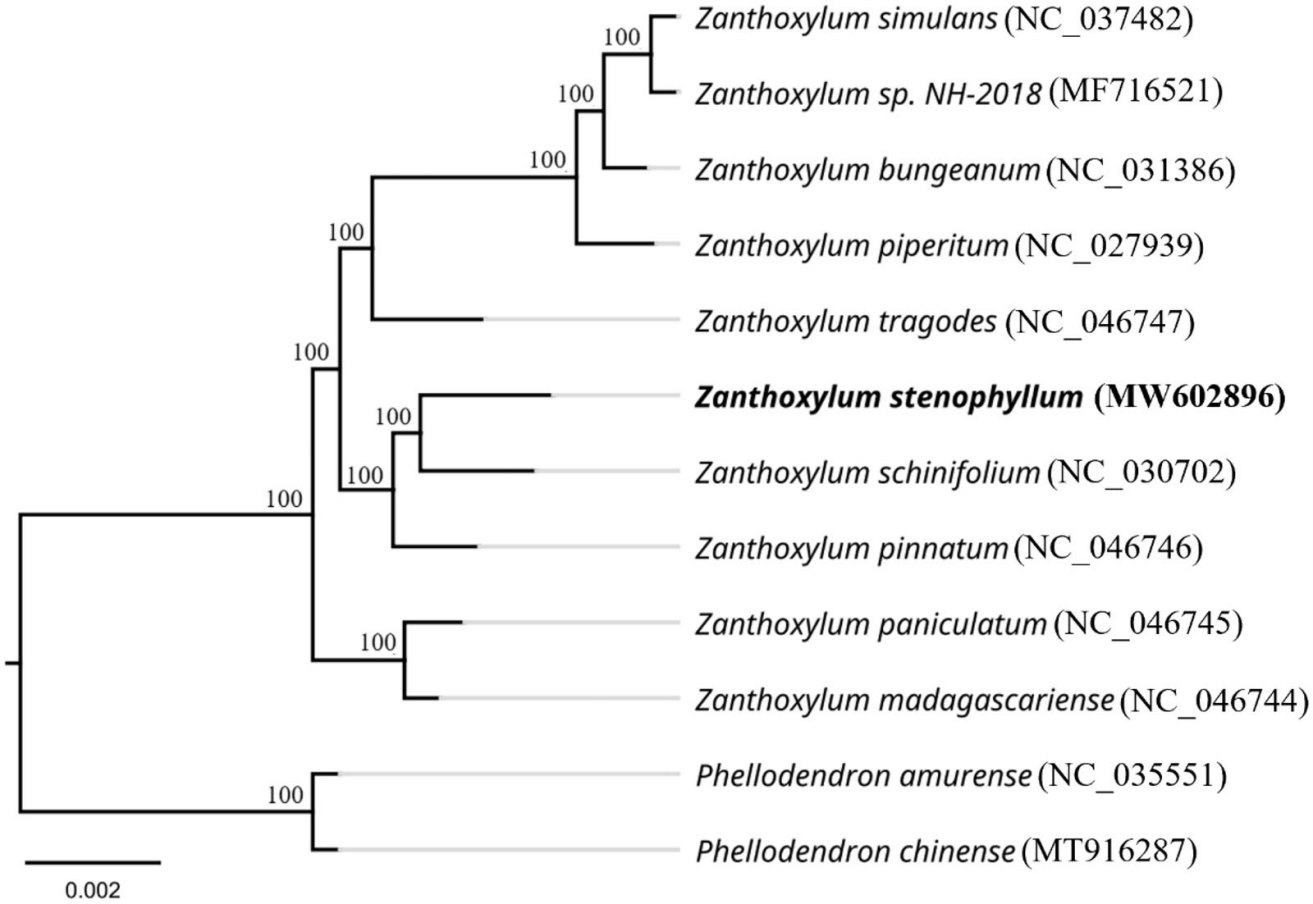
Maximum likelihood (ML) analysis of *Z. stenophyllum* and other related species based on the whole plastid genome sequence. *Phellodendron amurense* and *Phellodendron chinense* are the outgroup. Numbers near each node indicates ML bootstrap values.

## Data Availability

The genome sequence data that support the findings of this study are openly available in GenBank of NCBI at (https://www.ncbi.nlm.nih.gov/) under the accession no. MW602896/NC_058754. The associated BioProject, SRA, and Bio-Sample numbers are PRJNA680256, SRR17163938, and SAMN23766594, respectively.
